# The Effect of Different Implant Surfaces and Photodynamic Therapy on Periodontopathic Bacteria Using TaqMan PCR Assay following Peri-Implantitis Treatment in Dog Model

**DOI:** 10.1155/2018/7570105

**Published:** 2018-07-04

**Authors:** M. Madi, A. S. Alagl

**Affiliations:** Department of Preventive Dental Sciences, Periodontology Division, College of Dentistry, Imam Abdulrahman Bin Faisal University, Dammam, Saudi Arabia

## Abstract

**Introduction:**

Peri-implantitis is one of the late complications that leads to implant failure and is associated with specific microorganisms identified as periodontopathic bacteria. The objective of this study was to evaluate the relationship between the different implant surfaces and number of* Porphyromonas gingivalis, Tannerella forsythia,* and* Treponema denticola *using TaqMan PCR assay after peri-implantitis treatment using photodynamic therapy.

**Method:**

Forty-eight dental implants with four different surface treatments (M: machined; SA: sandblasted acid-etched; S: 1 *µ*m sputter HA-coated; and P: plasma spraying HA-coated) were inserted in six beagle dogs. After nine months of peri-implantitis induction, a split mouth design was used; on control side decontamination was performed using open flap mechanical debridement OFD with plastic curette, while photodynamic therapy PDT using diode laser (Ga Al As 830-nm) was used in the test side. For the following 2 weeks low-level laser therapy LLLT (10mW) was applied for the test side on alternative days for 6 sec on each implant side. Peri-implant microbial samples were collected using paper points and analyzed using TaqMan PCR before peri-implantitis treatment, immediately after treatment and 5 months posttreatment.

**Results:**

Both treatment modalities showed significant decrease in all bacterial count from baseline to immediately after treatment (P< 0.0001). The count increased between immediately after treatment to 5 months after treatment (P< 0.0001); however, the count after 5 months was significantly lower than at baseline. PDT had a stronger effect on reducing* P. gingivalis* count than T.* denticola* and* T. forsythi*a compared to OFD. For* T. forsythi*a, implant surface treatment had the greatest effect which was also statistically significant (P= 0.02) with considerably lower effect of PDT or their interaction.

**Conclusion:**

The results suggest that PDT and OFD have significant benefits in peri-implantitis treatment by reducing bacterial count. The presence of bacterial complex with different response to therapeutic modality suggests the use of combined decontamination methods for peri-implantitis treatment.

## 1. Introduction

The treatment of infectious diseases has become a challenging issue in implant dentistry [1]. Peri-implantitis is a plaque-induced disease that affects osseointegrated implants leading to progressive bone and soft tissue destruction. Peri-implantitis is considered as one of the factors that leads to implant loss and is associated with specific microorganisms identified as periodontopathic bacteria [2, 3].

Experimental as well as naturally occurring peri-implantitis bone defects have been commonly associated with supracrestal and an intrabony component [4]. Different surgical and nonsurgical modalities to treat peri-implantitis have been studied [5]. The aim of these treatment therapies was to eliminate bacterial contamination and to control peri-implant tissue inflammation together with arresting bone loss [6–9]. Contrary to nonsurgical therapy, open flap surgery was associated with better control and resolution of inflammation, together with new bone formation [1].

Photodynamic therapy (PDT) is a therapeutic method that uses photosensitizer and low-level energy source to target pathogenic bacteria [7]. The activation of the photosensitizer with appropriate wavelength leads to lethal changes to the target bacteria. This selective mode of action is considered the main advantage of PDT [10].

The influence of dental implant surface properties and treatment that favors bacterial biofilm formation in relation to peri-implantitis initiation and progression is still under investigation. Previous studies have demonstrated the association between periodontopathic bacteria and implant failure [11–13].

Mechanical or/and chemical decontamination of implant surface by have been suggested. Yet, the extent of bacterial residues that need to be eliminated in order to achieve a favorable outcome following treatment is still unknown [14]. Eliminating the bacteria from the rough implant surface is a challenging procedure due to the complex implant surface topography as well as its chemical constituents [15]. Surgical procedure to treat peri-implantitis and to decontaminate implant surface has been shown to be more predictable than nonsurgical method [13, 14]. The increasing bacterial resistance to antibiotics added to the demand for more specific methods for bacterial eradication like laser therapy [16, 17]. Bacterial elimination using laser has been investigated previously and is considered an alternative therapeutic modality in periodontal therapy [14]. PDT overcomes the drawback of using high power laser such as tissue damage, bone, or pulpal lesions. [16]

Immediately after placement, implant surface was coated with biofilm [18, 19]. Periodontal pathogens colonize the implant surface through plasma proteins present in the pellicle [20, 21]. Previous studies showed that implant surface roughness influences initial plaque formation. Moreover, larger numbers of* P. gingivalis w*ere observed to colonize titanium surfaces after serum coating, while larger number of* T*.* forsythia* were observed on titanium irrespective of the coating type [22, 23].

Qualitative diagnostic polymerase chain reaction PCR systems cannot precisely show the bacteria quantity only reflecting the presence of the investigated pathogen [24]. Thus, they have been shown to be unsuitable for evaluating treatment modalities in contrast to quantitative analysis methods that can efficiently evaluate the effect of treatment therapy. The TaqMan real-time PCR assay is a useful method for DNA quantitative recognition; it depends on the activity of* Taq *polymerase [25, 26].

The real-time PCR is more precise than conventional PCR to quantify specific bacteria that is associated with a disease or condition [27]. Determining the percentage of specific bacteria before and after treatment approach is considered a reasonable method to assess the proposed treatment. Lyons et al. [25] showed that relative quantity of a certain species in a mixed sample is more significance than determining the absolute number.

Thus, the current study was conducted to evaluate the effect of photodynamic therapy in peri-implantitis treatment on number of* P. gingivalis*,* T*.* denticola,* and* T. forsythi*a using TaqMan PCR assay.

## 2. Methodology

### 2.1. Animals

Animal care and surgical procedures were approved by the Animal Care and Use Committee of Tokyo Medical and Dental University. During the experiment, the dogs were fed once a day with a soft diet and water. In this study, six healthy beagle dogs (2 years old and weighing 11 to 12 kg) were used. Four weeks adaptation period was allowed before the initiation of the experiment. All surgical procedures were performed under general anesthesia, by using 0.1% medetomidine hydrochloride (Dormitor, Orion, Espoo, Finland) at 0.05 mL/kg as intramuscular premedication and 5.7% ketamine (Ketararu, Daiichi Sankyo, Tokyo, Japan) at 0.2 mL/kg (intramuscular). Infiltration anesthesia at the surgical site was performed with 2% xylocaine/epinephrine (DENTSPLY, Sankin, Tokyo, Japan) (1:80,000).

### 2.2. Surgical Procedures and Experimental Peri-Implantitis

Detailed description of the surgical procedures has been mentioned previously in Madi et al. [4]. Briefly, 12 weeks after bilateral extraction of all mandibular premolars, four dental implants were inserted bilaterally with different surface treatments (total of 48 implants) (M: machined; SA: sandblasted acid-etched; S: 1 *µ*m sputter HA-coated; and P: plasma spraying HA-coated). Healing abutments (3.5 x 3 mm) were connected to the implants and left to heal in a nonsubmerged position for 8 weeks. During the healing period 0.12% chlorhexidine-gluconate irrigation three times per week and scaling once per month were performed. Silk ligatures were inserted subgingivally around the implants to initiate peri-implantitis. Ligatures were removed when about 40% radiographic bone loss was observed then peri-implantitis progression was allowed for the following 5 months. Three weeks prior to treatment, oral hygiene procedures were performed.

### 2.3. Microbial Samples

Peri-implant bacterial samples were collected using paper points before, immediately, and 5 months posttreatment. Collection of bacterial samples was done from the peri-implant sulcus mesially and distally. First, partial isolation using cotton rolls and supramucosal debridement at the collection site was performed using sterile plastic curette. Four sterile paper points were then inserted into the peri-implant sulci until resistance was felt for 20 seconds [24]. All samples were collected by the same investigator and coded by a blinded assistant. The paper points were transferred into 200 ml cell lysis buffer and boiled at 100^0^C for 5 min and the supernatant was used as PCR template [28].

For each real-time PCR, 20 *µ*l of a mixture containing 1 *µ*l of lysed cells, 1xTaqMan Universal PCR Master Mix (Applied Biosystems), 200 nM (each) sense and antisense primer, and 250 nM TaqMan probe was placed in each well of 96-wells plate. Amplification and detection were performed using the ABI PRISM 7700 sequence detection system (Applied Biosystems)[26].

### 2.4. Treatment Procedures

**Figure 1:**
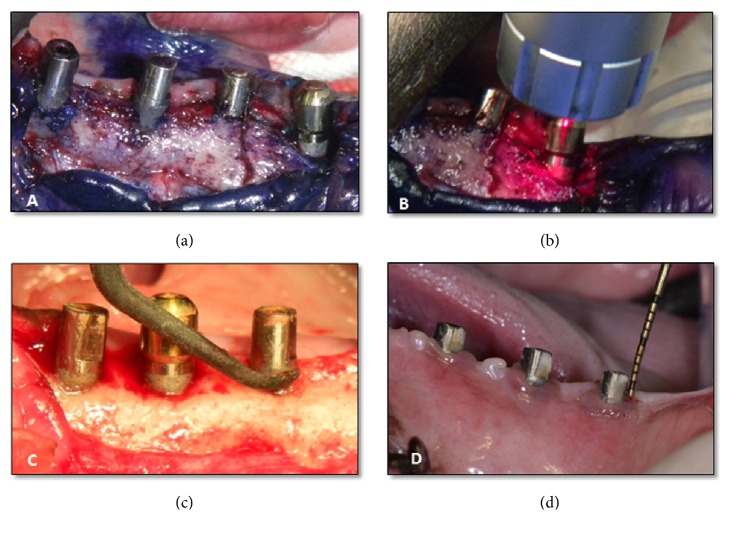
((a) and (b)) PDT using TBO and diode laser. (c) Open flap debridement. (d) Reduced probing depth and healthy gingival tissue was observed 5 months posttreatment.

Degranulation of the peri-implant defect was performed after reflecting a mucoperiosteal flap using plastic scaler (Implacare-IMPHDL6, Hu-Friedy, Chicago, IL). Using split mouth design, PDT was applied in one side (Figures 1(a) and 1(b)), while, on the other side, decontamination was performed after full thickness flap reflection by mechanical debridement using plastic curette OFD (Figure 1(c)).

In PDT group, toluidine blue O dye (Toluidine Blue O, Sigma, Poole, UK) at a concentration of 100 mg/mL was applied to the implant surface and the peri-implant defect for 5 minutes (Figure 1(a)). The stained area was immediately irradiated with a gallium aluminum arsenide Ga Al As 830-nm diode laser (LIGHTSURGE SQUARE 5, Osada) with a power output of 50 mW and an energy density of 4 J/cm^2^. The laser was applied to four surfaces of the implant (mesial, buccal, distal, and lingual) via a scanning method for 30 seconds on each surface (Figure 1(b)) [27, 29, 30]. For the following 2 weeks low-level laser therapy LLLT (10 mW) was applied for the test side on alternative days for 6sec on each implant side.

Chlorhexidine irrigation (0.12%) was performed three times per week for 5 months. Animals were sacrificed by overdose injection of sodium pentobarbital five months after treatment.

### 2.5. Clinical Evaluation

Peri-implant probing depth (PD) was recorded at baseline (before treatment) and at the time of sacrifice (after treatment) using a periodontal probe (Figure 1(d)). Measurements were performed from the mucosal margin to the bottom of the peri-implant sulcus at mesial, buccal, distal, and lingual aspects and the mean PD for each treatment group was obtained. Measurements were performed twice to ensure intraexaminer reproducibility and all duplicate measurements were within 5% the original measurements.

### 2.6. Statistical Analysis

Repeated measures analysis of variance with Huynh-Feldt correction to correct for departure from sphericity was used to assess the reduction in* P. gingivalis*,* T*.* denticola,* and* T. forsythi*a immediately and after 5 months controlling for implant types and treatment groups. Estimated marginal means were compared after adjustment using Bonferroni correction. The impact of group (PTD versus OFD) and implant treatment and their interaction was also assessed with calculation of p value and partial eta squared to quantify the effect.

## 3. Results

**Figure 2:**
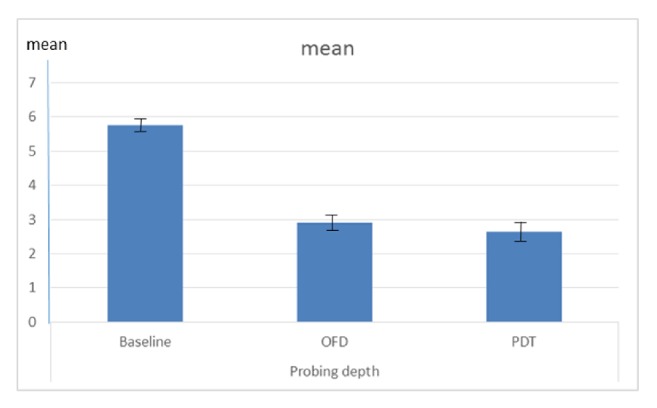
Mean change in probing depth at baseline and 5 months after treatment. Photodynamic therapy (PDT) and open flap debridement OFD groups.

**Figure 3:**
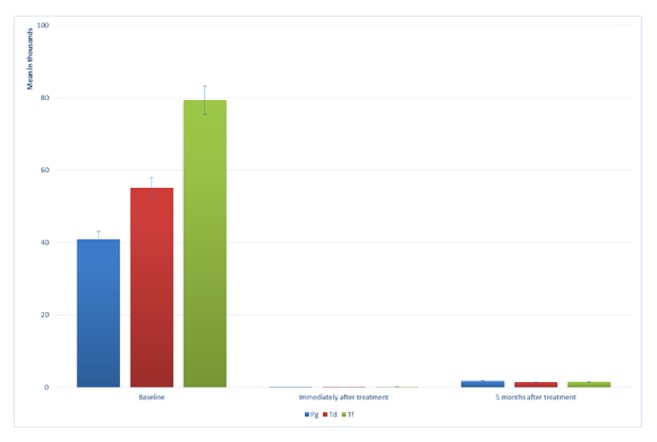
Change in bacterial count immediately after treatment and 5 months after treatment* Porphyromonas gingivalis *** Pg**,* Tannerella forsythia *** Tf, **and* Treponema denticola *** Td**.

Peri-implant probing depth (PD) showed a significant decrease after treatment for all implant types (P< 0.0001). No significant difference was observed between OFD and PDT group (Figure 2). A significant reduction in* P. gingivalis* count was observed immediately after treatment (P< 0.0001) and then slightly increased 5 months posttreatment (P< 0.0001). The count after 5 months was significantly lower than at baseline (adjusted mean at baseline= 40, 9667.7, SD= 2,533.9, mean immediately after treatment= 41.3, SD= 5.5 and mean after 5 months= 1,768.8, SD= 227.4). The same was observed in the case of* T. denticola* (adjusted mean at baseline= 55,200, SD= 2,204.5, mean immediately after treatment= 108.3, SD= 10.3 and mean after 5 months= 1,381.7, SD= 227.1) and with* T. forsythi*a (adjusted mean at baseline= 79,333.3, SD= 1,674.3, mean immediately after treatment= 143.8, SD= 18.4 and mean after 5 months= 1,450, SD= 81.7) (Figure 3).

The greatest effect on* P. gingivalis* count was that of PDT (partial eta squared= 0.15) with equally similar effect of implant surface treatment on its own or in interaction with PTD (partial eta squared= 0.07). The effect of implant surface treatment and its interaction with PTD on the reduction of* T. denticola* count (partial eta squared= 0.21 and 0.23) was greater than the minimal effect of PTD per se (partial eta squared= 0.003). For* T. forsythi*a, implant surface treatment had the greatest effect (partial eta squared= 0.44) which was also statistically significant (P= 0.02) with considerably lower effect of PDT or their interaction (partial eta squared= 0.06 and 0.07) (Table 1).

**Figure 4:**
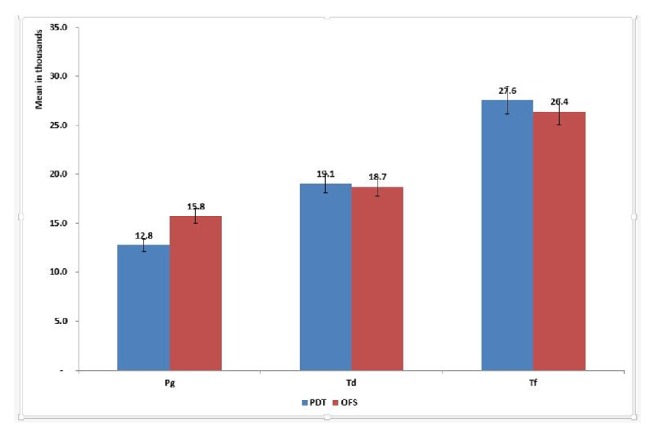
Mean bacterial count by implant surface treatment adjusted for the effect of time and use of PDT.* Porphyromonas gingivalis *** Pg**,* Tannerella forsythia *** Tf, **and* Treponema denticola *** Td**.

Regarding the impact of implant surface treatment on bacterial count and for use of PDT; no clear effect was observed in case of* P. gingivalis* count. In the case of* T. denticola* count, a stronger effect was noticed with the lowest count detected with machined and sputter HA-coated implant surfaces (adjusted mean= 17,280.4 and 17,718.7). The same pattern was also noticed with* T. forsythi*a where the lowest counts were detected in relation to machined (adjusted mean= 24,420.3) and sputter HA-coated surfaces (adjusted mean= 26,334.2) (Figure 4).

**Figure 5:**
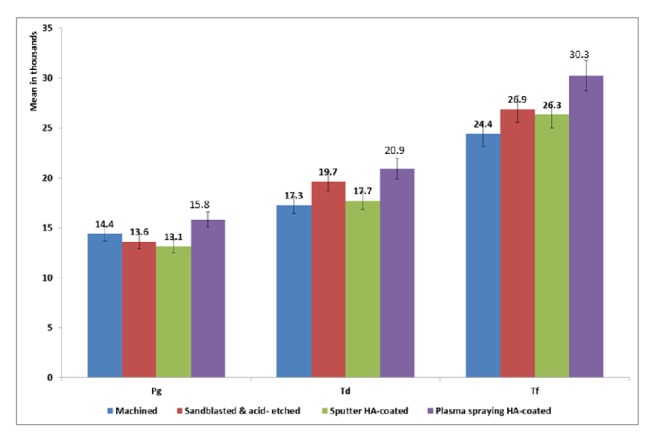
Overall estimated means by repeated measures ANOVA based on considering differences across time periods (baseline, immediately after and after 5 months, P<0.0001 for the 3 bacteria), by implant type (P=0.74 (Pg), 0.28 (Td), and 0.02 (Tf) ) and by surface treatment type (P= 0.11 (Pg), 0.82 (Td), and 0.0.34 (Tf)).

The current results showed that PDT had a comparable effect on* T. denticola* and* T. forsythi*a counts similar to OFS (adjusted means in* T. denticola* were 19,063.6 and 18,729.7 and in* T. forsythi*a were 27,562.6 and 26,388.9). By contrast, in case of* P. gingivalis* count, PDT had a stronger effect compared to OFS (adjusted means= 15,754.5 and 12,763.4). (Figure 5)

## 4. Discussion

Microbial profiles of peri-implantitis have been shown to include mainly the major periodontopathic microorganism with red complex bacteria as a predominant component (red complex:* Porphyromonas gingivalis* (Pg),* Tannerella forsythia *(Tf), and* Treponema denticola *(Td)) [31]. Similar to our findings, the increase in red complex count was associated with the shift from healthy to peri-implantitis state [32–35]. Similar to previous study [31], our results showed that* T. forsythia *was the most frequently found red complex organism in peri-implantitis sites followed by* T. denticola* and* P. gingivalis*.

Our results showed that the bacterial count was dramatically decreased immediately after peri-implantitis treatment in both groups in all implant types. These findings are similar to Hayek et al. [14] where* Prevotella sp., Fusobacterium sp*., and* S. Beta-haemolyticus* counts were significantly decreased after chlorhexidine irrigation and PDT. However, in their study* P. gingivalis *was not observed in peri-implantitis bacterial sample in contrast to our study in which red complex was detected. This could be due to the high sensitivity of the TaqMan PCR used in our study where in Hayek study they used bacterial culture.

In the current study PDT showed a bactericidal effect toward all bacterial types. However, PDT were shown to be more effective in reducing* P. gingivalis* counts regardless of the implant surface, while for* T. denticola *and* T. forsythia* implant surface treatment had influenced the reduced count to a greater extent, while PDT had a comparable effect to OFD in reducing* T. denticola *and* T. forsythia *count. This could reflect the need to a combined decontamination methods in treating peri-implantitis due to the presence of bacterial complex that differ in their response to a single therapeutic modality.

Previous studies [36, 37] showed that mechanical debridement with carbon curettes around implants had a favorable effect in peri-implantitis treatment. Similar to our findings, Gojkov-Vakelic et al. [38] showed the bactericidal effect of diode laser on periodontal pathogens. Meisel and Kocher [39] showed that periodontitis and peri-implantitis bacterial destruction following photosensitizing activation with laser. Gursoy et al. [40] reported that PDT can be beneficially used as antimicrobial agent. Shibli et al. [29] used PDT with 830 nm diode laser and guided bone regeneration for treating peri-implantitis on different implant surfaces. They observed a better bone gain in PDT group than control group.

*P. gingivalis* and* T. forsythi*a have been shown as the main pathogens in periodontal disease. Moreover, their interaction has been suggested to accentuate their virulence [21]. Fibronectin which is a main plasma protein has been shown to adsorb to Ti surface, thus facilitating adhesion of* P. gingivalis* [23]. Thus, plasma fibronectin acts as a mediator for* P. gingivalis* adhesion to Ti surfaces. Previous studies showed that the plasma fibronectin adsorption to Ti surfaces is roughness dependent [41, 42]. These come in agreement with our results in which less bacteria were detected on the machined implant followed by sputter hydroxyapatite surface compared to other implant types. Moreover, these could explain our findings in which bacterial interaction and bonding with the coated implant increase bacterial resistance to decontamination procedures. Thus, combined decontamination techniques (chemical and mechanical) can better reduce the bacterial count rather than a single decontamination method.

Previous study [43] observed a species-specific relation towards titanium and hydroxyapatite surface irrespective with serum coating or not. In the current study* T. denticola* and* T. forsythia *were more observed on acid etch and plasma HA-coated implants. Thus, material per se may affect the differences in bacterial adhesion. A previous study [44] showed that T.* forsythia* had high affinity to titanium than dentin and that its adhesion increases by presence of serum proteins.

In vivo and in vitro studies have been done to evaluate the bactericidal effect of photosensitizers and photodynamic therapy on periodontopathic bacteria [27, 45, 46]. Dobson and Wilson [47] showed that using toluidine blue O (TBO) and methylene blue (MB) were able to kill periodontopathic bacteria after exposure to He-Ne light. These come similar to our findings in which* P. gingivalis* count was significantly decreased following PDT for all implant types. Dörtbudak et al. [48] showed a significant immediate reduction in* A. actinomycetemcomitans, P. gingivalis*, and* P. intermedia *following photodynamic therapy and TBO application.

## 5. Conclusion

Within the limitation of this study, our results suggested that PDT and OFD have comparable effect in the treatment of peri-implantitis by reducing the bacterial count. The presence of bacterial complex with different response to therapeutic modality and implant surface treatment recommend the use of combined decontamination methods for peri-implantitis treatment.

## Figures and Tables

**Table 1:** Effect of implant surface treatment, PDT, and their interaction on the count of Porphyromonas gingivalis Pg, Tannerella forsythia Tf, and Treponema denticola Td.

	Effect of implant surface treatment			Effect of PTD			Interaction		
	F of repeated ANOVA	P value	Partial eta squared	F of repeated ANOVA	P value	Partial eta squared	F of repeated ANOVA	P value	Partial eta squared
Pg	0.43	0.74	0.07	2.79	0.11	0.15	0.39	0.76	0.07

Td	1.39	0.28	0.21	0.05	0.82	0.003	1.58	0.23	0.23

Tf	4.22	0.02*∗*	0.44	0.99	0.34	0.06	0.37	0.78	0.07

## Data Availability

The raw data will be provided upon request.
